# Glycemia Regulation: From Feedback Loops to Organizational Closure

**DOI:** 10.3389/fphys.2020.00069

**Published:** 2020-02-18

**Authors:** Leonardo Bich, Matteo Mossio, Ana M. Soto

**Affiliations:** ^1^IAS Research Centre for Life, Mind and Society, Department of Logic and Philosophy of Science, University of the Basque Country (UPV/EHU), San Sebastián, Spain; ^2^Institut d’Histoire et de Philosophie des Sciences et des Techniques, CNRS/Université Paris 1, Paris, France; ^3^Tufts University School of Medicine, Boston, MA, United States; ^4^Centre Cavaillès, République des Savoirs, CNRS, Collège de France et Ecole Normale Supérieure, Paris, France

**Keywords:** organicism, feedback loop, organizational closure, glycemia regulation, proof of concept (POC), functional constraints

## Abstract

Endocrinologists apply the idea of feedback loops to explain how hormones regulate certain bodily functions such as glucose metabolism. In particular, feedback loops focus on the maintenance of the plasma concentrations of glucose within a narrow range. Here, we put forward a different, organicist perspective on the endocrine regulation of glycaemia, by relying on the pivotal concept of closure of constraints. From this perspective, biological systems are understood as organized ones, which means that they are constituted of a set of mutually dependent functional structures acting as constraints, whose maintenance depends on their reciprocal interactions. Closure refers specifically to the mutual dependence among functional constraints in an organism. We show that, when compared to feedback loops, organizational closure can generate much richer descriptions of the processes and constraints at play in the metabolism and regulation of glycaemia, by making explicit the different hierarchical orders involved. We expect that the proposed theoretical framework will open the way to the construction of original mathematical models, which would provide a better understanding of endocrine regulation from an organicist perspective.

## Introduction

In recent years, an increasing number of contributions in theoretical biology and philosophy have been advocating an organicist perspective (Gilbert and Sarkar, 2000; Etxeberria and Umerez, 2006; Soto and Sonnenschein, 2006; Moreno and Mossio, 2015; Soto and Sonnenschein, 2018). According to organicism, theoretical and experimental biology – and notably physiology – should address aspects of living systems in light of their integration into a coherent unit, understood as a natural system endowed with a distinctive complexity^1^.

One of the fundamental notions of organicism is ‘organization,’ a concept more specific than a mere synonym of ‘configuration’ or ‘arrangement,’ which relies on a rich theoretical tradition inspired by the work of Kant (1790) and Bernard (1865) and further developed in the 1960’s and 1970’s (Piaget, 1967; Rosen, 1972; Pattee, 1972; Varela et al., 1974; Gánti, 1975). We use the term ‘organization’ to refer to a certain mode of interaction between the parts of a system, distinctively realized by biological organisms when compared to other kinds of natural systems or to artifacts. In a first approximation (see Section “Organizational Principles” for more details), organization refers to a regime in which (1) a set of parts are related to each other so as to constitute a system that displays both functional differentiation and integration; (2) the activity of the whole system plays a role in producing and maintaining its parts over time: organized systems maintain themselves.

Although organicism is gaining momentum in the theoretical literature, a wider reception in biology would be achieved if its applications were shown to improve experimental and modeling practices. In the context of the issue topic “Multilevel Organization and Functional Integration in Organisms,” the general objective of this investigation is to propose a “proof of concept,” an illustration of how organicist – and more precisely, organizational – principles can advantageously modify biological modeling. We do so by focusing on a particular case study: the regulation of plasma glucose concentrations (glycemia) in mammals.

Our aim is to show that the representation of this phenomenon substantially changes when shifting from a standard characterization in terms of feedback loops to an original one grounded in organizational principles. Feedback loops are control devices that, although not specific to biology, have become since the 1930’s an important tool in different areas of biology (e.g., neurophysiology, see for instance Lorente de Nó, 1934), often in association with the idea of homeostasis (Cannon, 1929). Since then, they have been used to model and understand dynamically stable situations in which the value of a variable appears to be actively maintained within a given range (Wiener, 1948).

Feedback loops also provide a useful description of certain complex physiological control phenomena. In endocrinology, the concept of negative feedback plays a central role in the understanding of the maintenance of calcium and glucose plasma concentrations within a narrow range (Widmaier, 1992; Wilkin, 1997; Mundy and Guise, 1999; Carmeliet et al., 2003). Positive feedback also plays an important role in endocrinology although, in contrast to the negative feedback, it is used to explain how an effect is amplified by creating instability, such as the positive oxytocin loop of parturition and the estrogen-triggered positive feedback of ovulation (Higuchi and Okere, 2002; Russel et al., 2003; Christian and Moenter, 2010).

In spite of their widespread and practical use in developing dynamical models of how physiological variables are maintained as stable, we claim that feedback loops might bring about a weakening of biological explanation. In particular, the description of glucose regulation – the phenomenon on which we focus here – in terms of feedback has three main problematic implications: first, it tends to neglect the nature of the relations between the parts and their place within the whole organism; second, it flattens the description of the system by overlooking the various categories of objects in play as well as their hierarchical relations; third, it is built on the relationship between concentrations of glucose, insulin and glucagon, and does not foster the inclusion of further factors involved in the regulation of glucose metabolism, such as the nervous system and the gut. In contrast, we posit that the organicist perspective promotes a more specific understanding of how biological organisms realize homeostatic behavior in relation to certain variables. Biological homeostasis is derived from more fundamental organizational principles and explained as a result of the regulatory capacity of a functionally differentiated hierarchical biological organism.

It is worth underscoring that our goal here is not to provide a full-fledged model of the regulation of blood glucose concentration, although we do provide some preliminary guidelines. Rather, we aim to show how organizational principles allow us to take into account the characteristic complexity of biological systems when focusing on a specific phenomenon and, therefore, open the way to the elaboration of richer and more appropriate models.

## The Standard Representation of Glycemia Regulation: the Feedback Loop

The model of homeostasis through negative feedback has a long history. It dates back to the introduction of the notion of “conservation of the internal milieu” by Bernard (1865) in the 19th century and Cannon’s (1929) subsequent introduction of the concept of homeostasis in the early 20th century. Negative feedbacks were explicitly formalized by cybernetics (Wiener, 1948) and traditionally used in engineering in the 1960’s (see Lewis, 1992, chapter 1). Standard accounts of feedback can also be found in the classical literature of systems theory (see for example Rosen, 1968, p. 37; Jones, 1973, p. 80).

A negative feedback describes phenomena in which the value of the output of a system is used to modify the activity of the system (the output “feeds back”) in such a way as to create a loop that reduces fluctuations in the output itself, and to make its value homeostatic within a specific range. According to Rosen (1968, p. 37), a feedback loop is characterized by the presence of a controller and a controlled system: “The relation of the controller and the controlled system is the following: the inputs to the controller are the original inputs to the system, together with the outputs of the controlled system; and the inputs to the controlled system are the outputs of the controller. Thus, in effect the controlled system is supplied with a new input set, determined partly by its own past activity; i.e., we have a system with a feedback.” As mentioned, feedback loops are widely used to model homeostasis in numerous biological fields. Although they share the core idea, different models vary regarding the number and kind of elements constituting the feedback system. In what follows, we rely on a recent characterization provided by Modell et al. (2015), which gives a general and complete description of the feedback loop, and is considered as a standard for education purposes in physiology. While most feedback models usually focus on the relationship between a reduced set of variables, this description has the advantage of making explicit *all* the elements that are often left implicit.

**FIGURE 1:**
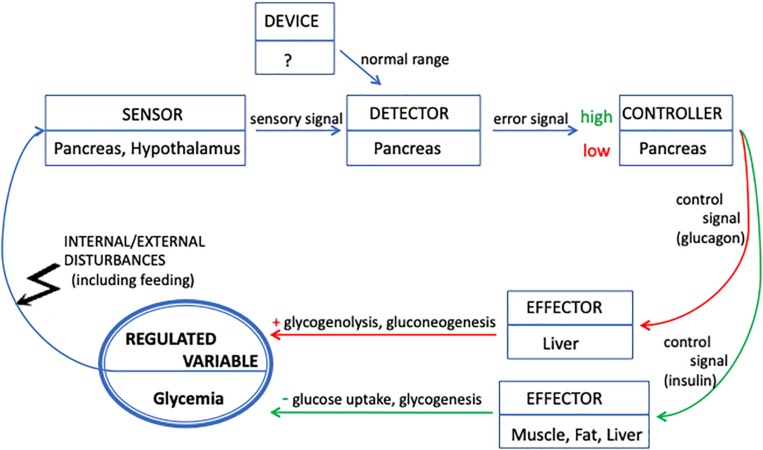
Representation of glycemia regulation in terms of a feedback loop. The regulated variable (glycemia) is represented by the bold circle. The functional components involved in the feedback loop are represented by rectangular boxes indicating their localization in the organism. Blue arrows indicate interactions between components. Red arrows indicate interactions (signals and metabolic processes) that, triggered by low glycemia, produce an increase in glucose concentration. Green arrows decrease the glucose concentration as a response to high glycemia. The system feeds back into itself by sensing the value of glycemia, which is the target of its regulatory activity. The feedback loop maintains the value of glycemia as homeostatic by modulating the release of insulin and glucagon on the basis of the difference between the actual value and the normal range.

There are five components that a system must contain in order to realize a feedback loop and to maintain a target variable – the “regulated” one – as homeostatic, i.e., within a specific interval (see Figure 1). Paraphrasing Modell et al. (2015), these are:

(1) A *device/mechanism* that establishes the *normal range* (“set point”) of values for the regulated variable;
(2) A *sensor* that measures the actual value of the regulated variable and emits a *sensory signal*;
(3) A *detector* that compares the actual value (through the sensory signal) with the normal range. The result of this comparison is an *error signal* that is sent to the controller;
(4) A *controller* that interprets the error signal and determines – through a *control signal* – the value of the outputs of the effector;
(5) An *effector* that responds to the *control signal* and modifies the value of the regulated variable.

As Modell et al. (2015, p. 261) explain: “Such a system operates in a way that causes any change to the regulated variable, a disturbance, to be countered by a change in the effector output to restore the regulated variable toward its set point value. Systems that behave in this way are said to be negative feedback systems.”

Let us apply this description to the case of glycemia regulation and locate the components of the feedback loop within the organism. The description of glycemia homeostasis should show how the organism manages to maintain a steady supply of glucose, regardless of whether it is feeding or fasting. Glucose, the *regulated variable*, is the principal source of energy for the organism in general, and particularly for the brain.

During fasting, the liver (the main *effector*) breaks down stored glycogen, and glucose is secreted into the bloodstream. The activity of the liver is modulated by hormones that, in the terminology of feedback control, are said to carry signals. Glucagon, a hormone produced by the pancreas, increases glycogenolysis and the synthesis of glucose from amino acids and lipids (gluconeogenesis) in the liver. In turn, the increase of glycemia triggers in the pancreas the release of insulin, the hormone that facilitates the entry of glucose into the muscle compartment (the other *effector*) where it is needed for physical activity and inhibits glucagon secretion. Muscular uptake decreases glycemia, which induces the pancreas to secrete more glucagon, and so forth (see Figure 1). In this picture, that focuses on the role of hormones in modulating the effectors’ activity, the role of *sensor* is supposed to be played by chemosensors located in the pancreas and hypothalamus, structures that also play the role of *controllers*. In contrast, the *detector* is not clearly distinguished and located; however, it is inferred to be located in the pancreas. A very important point here is that the rate of secretion of hormones, as well as their effect in controlling glycemia, is not an “all or none” phenomenon. That is, glucose, insulin and glucagon are always present in the bloodstream.

Feeding leads to the storage of glucose as glycogen. More precisely, food intake, digestion and absorption increase glycemia in the systemic circulation that reaches all organs; in particular, the increase is massive in the portal blood that supplies the liver. High glycemia is sensed by the pancreas that, by also playing the role of *detector* and *controller*, triggers a response mediated by its beta-cells. These cells release insulin into the bloodstream. Insulin promotes the transport of glucose into cells, predominantly those in skeletal muscle and adipose tissue (both tissues are *effectors*), and its conversion into glycogen in these compartments as well as in the liver. As a result, glycemia decreases.

In both situations, a negative feedback is said to occur between low glycemia and insulin concentration, because pancreatic beta-cells slow down the rate of insulin secretion when glycemia is low, which results into the stabilization of glycemia at some low concentration. Additionally, a second and simultaneous chain of events takes place. Low glycemia, stabilized by the decreased rate of insulin release, suppresses the inhibition of alpha-cells, which increases the rate of glucagon release. Glucagon promotes the hydrolysis of glycogen in the liver and release of glucose in the blood, which increases glycemia. This set of coupled processes generates a homeostatic situation, in which glycemia is maintained within a viable range by compensating for both decreases and increases of glycemia. During both fasting and the intestinal absorption of sugars, the circularity of glucose homeostasis is given by the fact that, starting from normal glycemia, variation in glucose concentration results in the restoration of normal glycemia. Modell’s description of the feedback system now applied to the control of glycemia is shown in Figure 1.

The diagrammatic representation of glycemia regulation in terms of feedback loops serves the purpose of focusing on and making explicit the circular relations between coupled variables (the concentrations of glucose, insulin, and glucagon) that are relevant to explain the homeostatic behavior of the organism, in relation to one of these variables, namely, glycemia. The negative feedback diagram provides a useful tool for global evaluation of the ability of the organism to blunt the increase of blood glucose concentrations after administering a glucose overload. Indeed, the glucose tolerance test provides a measure of the ability of the organism to regulate glycemia and thus to evaluate normalcy, pre-diabetes and diabetes.

Yet, in spite of the descriptive role and clinical usefulness of the feedback loop, we submit that such a representation does not foster an adequate understanding of biological organization because most functional, topological and hierarchical features of the organism remain hidden. What is put forward is the relation between variables, without revealing much about the complexity of the organism that should be spelled out to better understand how it controls its own glycemia.

We think that the inadequacy of feedback loops takes four forms. First, they tend to favor the idea of a neat localization of functional components. While it usually works for manmade machines because their parts have been designed separately and assembled, a neat localization applies much less clearly for organisms, in which a given function can be jointly performed by several components and a given component or structure can perform different functions. In addition, some functions can be distributed over the entire system and thus are non-localizable. Second, feedback loops tend to represent the system as a *flat* chain of interacting components. As the above diagram illustrates, the system is described as a set of functional components realizing a chain of steps, with no hierarchies or distinction of levels. Although they perform different functions, each component interacts in the same way with the following one in the chain, by either activating or inhibiting its activity (be it through a signal or not), following the kind of perturbation affecting the system. In this respect, there seems to be – to use a philosophical expression – only one kind of “causal relation” at work in a feedback loop, which makes us claim that the resulting representation flattens the characteristic complexity of the system. Third, feedback loops do not foster the search of additional components and variables that might play a role in the homeostatic behavior. Of course, feedback diagrams can be enriched by new empirical knowledge; yet, they focus exclusively on the relation between several variables (in our example, the concentration of glucose, glucagon and insulin) so as to understand the stability of the variable of interest (glucose). Accordingly, they ignore – and do not encourage exploring – the relationship between these variables and other physiological components which converge in diverse ways to control the concentration of glucose. Fourth, a description in terms of feedback assumes the existence of a value (or, more precisely, an interval of values) to be kept stable – a set point – without providing an explanation of how it is established or how it can be modified.

The epistemological stance that lies behind these weaknesses, we submit, is the classical cybernetic analogy between organisms and machines, which are supposed to realize the same kind of control capacities. Although some aspects of biological organisms can certainly be described in a way commonly used to describe machines, the analogy can be misleading in several ways as it risks concealing crucial differences between the two classes of systems. In particular, this holds for homeostasis, whose description in terms of feedback emphasizes the common capacity of organisms and machines to maintain a given variable as stable while overlooking biological specificities. While in machines homeostasis is a goal determined by an external designer, in organisms it constitutes a means to achieve the more fundamental goal to maintain oneself as alive. Contrary to machines, organisms maintain some variables as stable only insofar as this promotes their self-maintenance, which means not only that homeostasis is achieved in a different way, but also that alternative behaviors and variations can be observed in some circumstances. In this respect, a fundamental difference between organisms and machines points to the device that establishes the normal range of the regulated variable. In machines the normal range, set by the human designer, is recorded in some component of the system, while in organisms, as mentioned above, it is unclear what process sets the normal range (many biologists would presumably appeal to evolution). Furthermore, it is highly debatable whether the normal range is recorded in some specific component of the system (Fitzgerald and Bean, 2018). While feedback loops are blind to these differences, we submit that the organizational framework fosters a more adequate understanding of organismal homeostasis (and specifically glucose homeostasis) as a manifestation of distinctive biological capacities.

## Organizational Principles

The theoretical framework built on the notion of organization characterizes biological systems as autonomous, i.e., endowed with the distinctive capability to constantly produce, transform, repair and replace their own components, and maintain themselves through exchanges of matter and energy with the environment (Piaget, 1967; Rosen, 1972; Varela et al., 1974; Kauffman, 2000; Moreno and Mossio, 2015). Unicellular and multicellular autonomous systems locally oppose the increase of entropy and the thermodynamic tendency toward equilibrium. They maintain themselves in far-from-equilibrium conditions – i.e., in highly improbable dynamic distributions of energy and matter – by *controlling* the thermodynamic flow.

Biological control can be defined as the capacity to modify the dynamics of a system toward a certain state (e.g., an enzyme acting upon concentrations of metabolites, see Rosen, 1970; Fell, 1997). It implies an asymmetry between the controller and what is controlled^2^. In biological systems control is exerted by molecular and supra-molecular structures (such as enzymes or membranes), by cells, extracellular structures, tissues and organs that are produced and maintained by the system itself. These structures act as *constraints* on the thermodynamic flow. A constraint is a structure that has a causal effect on a process (or transformation) while being locally unaffected by the process at the time-scale in which it takes place (Montévil and Mossio, 2015). Constraints play the role of local boundary conditions that enable specific processes to take place by reducing their degrees of freedom^3^ (Pattee, 1972; Kauffman, 2000). By doing so they locally channel the flux of energy and matter, chemical reactions, etc. toward outcomes that can contribute to the functioning of the system, and that would be extremely improbable (or practically impossible) in the absence of such constraints. A paradigmatic example of a biological constraint is an enzyme that, by lowering the activation energy necessary for a reaction, catalyzes it toward an otherwise improbable product, which in turn can be employed to perform some functional activity for the system. Another example is given by the structures constituting the vascular system, which constrain the circulation of oxygen to the neighborhood of cells, where it participates in respiration. As a matter of fact, it has been argued that any biological structure or part to which biologists ascribe a *function* can be conceptualized as exerting a constraint on a process or transformation (Mossio et al., 2009).

The specificity of living systems is that they are *organized*, by which we mean that their constitutive constraints collectively produce and maintain each other and, ultimately, the whole system itself. The resulting organization realizes a distinctive regime, called *organizational closure* or *closure of constraints*, in which the very existence and activity of a set of constraints depends on their mutual relations and interactions (Moreno and Mossio, 2015; Montévil and Mossio, 2015). Unlike other natural self-maintaining systems such as dissipative structures – which are spontaneous, are mostly or fully determined by external boundary conditions and emerge anew each time under specific environmental conditions – living systems are historical and vastly contribute to determine their own conditions of existence (Mossio and Bich, 2017).

**FIGURE 2:**
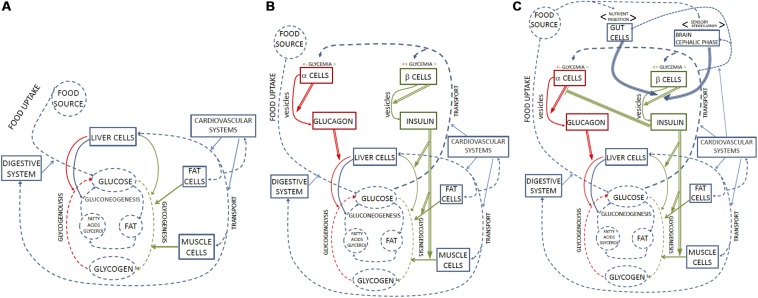
Representation of glycemia regulation in terms of organizational closure. **(A)** Represents the first-order functional regime, in which a set of functional parts (rectangular boxes) constrain (blue arrows) processes and transformations (dotted blue arrows) involving glucose as a product or metabolite. Metabolic substrates and products are in dotted circles. Constraints and processes in red and green are those that affect glucose and glucagon concentrations. Organizational closure is realized by the fact that each part acting as a constraint is also the product of a metabolic process within the system. As a result, the system self-maintains. **(B)** Includes second-order regulatory functions to organizational closure. The regulatory subsystem comprises parts which play the role of constraints that are sensitive to glycemia (sensitivity to *x* is marked by <*x*>above the pertinent box) and second-order constraints (double arrows), which modulate the activity of first-order ones. Regulatory constraints marked in red are sensitive to low glycemia and accelerate glycogenolysis; regulatory constraints marked in green are sensitive to high glycemia and accelerate glycogenesis. The overall result of regulation is the homeostasis of glycemia, which is not represented as a separate ‘component’ in this figure, but constitutes an effect of the biological dynamics controlled and regulated by different orders of organized constraints. **(C)** Adds third-order regulatory functions. The regulatory subsystem includes parts that play the role of constraints sensitive to food (either by ingestion or perception) and exert third-order constraints (triple arrows) on second-order constraints. The inhibitory action of insulin on alpha-cells is also interpreted as a third-order constraint. Second- and third-order regulatory constraints are also the target of metabolic processes, which contribute to their maintenance: therefore, the whole hierarchical system realizes organizational closure.

At the intracellular level, the coordinated activity of organized constraints such as proteins, membranes and nucleic acids, contributes to the realization and maintenance of the organized system that contains them, by channeling the flow of matter and energy necessary to build these components and to run the internal processes of the system (Ruiz-Mirazo and Moreno, 2004). Multicellular systems, while constraining thermodynamic processes to sustain their own metabolism, also exert control upon the activity of the cells that constitute their tissues and organs (Arnellos et al., 2014; Longo et al., 2015; Montévil et al., 2016; Soto et al., 2016; Veloso, 2017; Bich et al., 2019). At both organizational levels, the set of mutually dependent control constraints is responsible for the realization of what we label the *first-order regime of closure^4^* (Figure 2A).

Yet the first-order regime is only one dimension of the living organization. In fact, one of the distinctive features of biological autonomous systems is their adaptivity, and in particular the capacity to modify their own first-order regime in relation to changes in internal needs and external conditions. Such a capacity can take two main forms. On the one hand, the first-order regime, while performing metabolic activity, exhibits some dynamic stability that makes it capable of compensating *as a network* for small perturbations, mostly stoichiometrically^5^ by means of changes in concentrations (for example, by relying on the balance between supply and demand of metabolites). In such cases the network is maintained by the same attractor or shifts toward a new one among those available (in case of multi-stable networks).

On the other hand, living systems possess a specialized class of organized constraints (which means that they are also maintained by the organism), that we label *regulatory*, that act as higher-order controllers upon first-order constraints. Regulatory constraints modulate the first-order regime of closure in response to specific variations induced by external perturbations, but also by internal dynamics of the organism (Bich et al., 2016). They do so by selectively inducing shifts between distinct available physiological^6^ or agential regimes^7^. A typical example is the regulation of the direction of movement in bacteria performing chemotaxis, which depends on the sensing of sugar gradients and is achieved by shifting the motion of the flagellum between tumbling and rotating^8^. Another example of dynamics requiring regulation is protein synthesis. Not all possible proteins can be available simultaneously in the system, due to spatial and energetic limitations; therefore, the various components should be synthesized just when they are needed. Also, some subsystems may work differently and with different requirements, not always compatible, and their operations need to be modulated in such a way that they can jointly contribute to the maintenance of the system while avoiding potential conflicts (Bich, 2018)^9^.

Regulation provides the organism with the possibility of acting upon its own dynamics. Conceptually, a regulatory subsystem includes a set of dynamic constraints operating in a way that is distinct from first-order constraints, and *collectively* satisfying two main requirements:

(1) The presence of constraints that act as second-order controllers, which means they modulate the activity of other constraints in the system, instead of directly channeling metabolic processes (which is what first-order constraints do)^10^;
(2) The presence of constraints that, by being specifically sensitive to variations in internal or external conditions, begin to perform a qualitatively different function and, thereby, bring about the activity of the regulatory subsystem.

The first requirement implies that there is no regulation when the change of first-order dynamics is not due to the action of a constraint but, instead, to a variation in the concentration of a metabolite, which changes the rates of the metabolic pathways because of the effects of the law of mass action. The second requirement, in turn, means that regulation is a context-sensitive phenomenon, which is triggered by specific conditions, as for example when an enzyme reacts to an allosteric interaction. In particular, there must be constraints that perform a function only when a given internal or external variable (to which they are “sensitive”) undergoes a specific change^11^ (Figure 2B).

For regulation to be functional in the context of the organism, there must be a connection between what stimulates the regulatory subsystem and what this subsystem acts on. It means that the regulatory action should make the first-order dynamics viable in the context that activated regulation in the first place. For instance, there is a relation between blood glycemia and glycogenesis, so that a modulation of the latter constitutes an adaptive response to the viability challenge raised by a significant variation of the former. Yet, this relation does not take the form of a direct dependence, which means that variations of the conditions that activate a sensitive constraint (e.g., through an allosteric interaction) affect the subsystem differently than variations of the processes on which second-order constraints exert their control^12^. This asymmetrical relation is what we have referred to elsewhere as “dynamical decoupling,” between regulatory and regulated constraints (Bich et al., 2016), by which the regulatory subsystem exhibits degrees of freedom that are not specified by the dynamics of the regulated one. Such a local independence allows regulatory subsystems to modulate first-order constraints in a relatively autonomous way.

In brief, regulatory subsystems exhibit context-sensitive activity^13^ that adaptively modulates metabolic processes by acting upon first-order constraints. They act when specific conditions are met, so as to maintain the overall viability of the system. Let us apply these ideas to the case of glucose regulation.

## Reinterpreting Glucose Regulation From Organizational Principles

How do organizational principles guide the elaboration of a biological model? To study a phenomenon, the organizational framework requires the identification of the relevant processes, the time scales at which they can be described, and the first-order constraints acting upon them at those times scales. Once these objects are identified, the next step consists of determining the dependencies between the constraints so as to obtain a closed graph, in which at least a subset of constraints are maintained by processes under the control of other constraints, so that the entire network can be said to realize collective self-maintenance. Moreover, if the aim is to understand how this network of constraints is modulated in response to external perturbations or changes in the internal state of the system, second-order dynamically decoupled constraints need to be identified and integrated into the closed graph of dependencies^14^.

Let us illustrate how this general procedure can be applied by considering the case of glycemia regulation in multicellular systems, specifically mammals. Glucose *as a metabolite* constitutes a primary energy source for these organisms. The plasma concentration of glucose depends on food uptake and on those internal metabolic processes (as well as organismal activities, such as exercise) that consume, transform, store and release this sugar. As shown in the feedback loop diagram, glycemia is kept within a specific interval in the blood despite variations affecting the supply and consumption of glucose. From an organizational perspective, such a homeostatic behavior is achieved through the contributions of both first-order compensatory capacities and higher-order regulatory functions, the latter ones involving different organs and resulting in the modulation of the whole first-order metabolic regime.

To describe the regulation of glucose metabolism that results in homeostasis, it is first necessary to identify the key processes involving glucose as a metabolite and the first-order constraints that control it. This first-order regime is a theoretical extrapolation, because there is no real organism devoid of regulatory constraints. However, experiments removing the constraining action of insulin and glucagon in mice give us a “real-life” approximation of how the first-order regime would work in the absence of the main regulatory constraints (Unger and Cherrington, 2012). As we will see, some of these first-order constraints are responsible for the presence of glucose in the blood, which makes it available to all cells, others for its removal from the bloodstream for utilization or storage. Glucose metabolism consists of different processes, which are:

(1) Glucose *uptake* by the cells of different tissues (brain, intestine, liver, etc.), constrained by glucose transporters in the cell membrane;
(2) Food *intake*, which includes the ingestion and digestion of carbohydrates and drastically changes the amount of glucose in the system. It is constrained by the digestive system (its dynamic structural constraints, the digestive enzymes and the absorption by the epithelial cells of the intestine). Food intake is the main source of variation for the first-order regime of utilization and production of glucose;
(3) Intracellular *glycolysis*, the breaking down of glucose molecules into pyruvate as part of the process of production of ATP. This process is constrained by enzymes in the cell metabolism;
(4) *Glycogenesis*, which consists of the transformation of glucose into glycogen for storage: a process constrained mainly by liver cells, striated muscles cells and cells of the white adipose tissue in which it is then stored;
(5) *Glycogenolysis*, the transformation of stored glycogen into glucose, constrained by all cells that store glycogen. Glycogenolysis by hepatic cells is the main source of blood glucose during fasting;
(6) *Gluconeogenesis*, also constrained by liver cells, which produce glucose anew starting from amino acids, lipids, pyruvate and lactate;
(7) Glucose *transport*, responsible for the distribution of glucose in the system: once this sugar reaches the bloodstream (after its absorption in the intestine, or after its secretion by the liver during fasting) its distribution in the body is constrained by the vascular system (e.g., the portal vein for transport to the liver, the hepatic veins for release into the general circulation).

All the constraints acting on the previous processes (namely: the digestive system, the vascular system and different organs and cell types) are in turn maintained by the glucose supply they constrain. By controlling processes involving glucose, they therefore contribute to their own conditions of existence and to the overall maintenance of the biological system that harbors them. In sum, they contribute to the realization of a regime of first-order closure (see Figure 2A).

How does the system manage changes in glucose concentration? In basal conditions, in which there is neither an abrupt intake nor a high need for glucose, the organism is constantly subject to small variations in glycemia due to stochastic interactions and the dynamic balance among the first five coupled processes. This first-order regime exhibits dynamic stability with respect to such variations mainly through a balance of processes 4 (glycogenesis) and 5 (glycogenolysis), plus 3 (glycolysis)^15^. Supply and demand effects such as a small increase in glucose concentrations in the blood, or of glycogen content in the cells, can speed up, dampen or inhibit the processes that rely on or produce these metabolites. For example, the glycogenesis pathway in muscle cells is characterized by two steps: the phosphorylation of glucose into G6P, constrained by the transport/hexokinase (GT/HK) subsystem; and the production of glycogen from G6P, constrained by the enzyme glycogen synthase (GSase). Due to both allosteric interactions and the effect of mass action, an increase in the supply of glucose can favor these reactions, while the accumulation of glycogen in turn inhibits the pathway. Because of these network properties the first-order regime achieves – with no regulation in the organizational sense – a balance that allows it to compensate for small variations in glycemia and at the same time exert a tight control upon this and other individual pathways (Figure 2A).

Such a delicate balance of first-order control mechanisms is however insufficient to maintain glucose homeostasis in the organism, both in basal conditions and – a fortiori – in response to greater alterations of glycemia caused by food intake or sudden high energy needs^16^. This is why regulatory constraints intervene to selectively modulate the first-order constraints in a coordinated way. By doing so, they allow the metabolism to speed up glycogenesis and inhibit glycogenolysis in the presence of high glucose concentrations, and vice versa in the case of low concentrations. A central role in glucose regulation is played, as discussed in Section “The Standard Representation of Glycemia Regulation: The Feedback Loop,” by the hormones insulin and glucagon released by the pancreas. From an organizational perspective, they can be treated as *second-order* constraints that modulate the functioning of other first-order constraints. Insulin does so in three ways: it facilitates glucose uptake in muscle and adipose tissue; it increases glycogenesis in liver, striate muscle and white fat cells; and it inhibits glucagon secretion by pancreatic alpha-cells. Glucagon, in turn, increases glycogenolysis and gluconeogenesis in the liver, and thus the release of glucose in the bloodstream.

Let us follow the conceptual scheme introduced at the end of Section “Organizational Principles” and focus first on the activation of the regulatory subsystem. Pancreatic beta-cells continuously produce and store insulin in vesicles and secrete it into the bloodstream. The secretion of insulin occurs when the insulin containing vesicles already present in the cells fuse with the cell membrane and release the hormone. In basal conditions, the release of insulin is low, but when the glycemia rises, beta-cells secrete it abundantly, thus modulating the metabolic activity of the first-order regime. Note that the activation of regulation is independent of insulin synthesis and the subsequent glycolysis, which is consistent the idea of dynamical decoupling discussed above. As a matter of fact, as we discuss just below, the activation does not even depend directly on glucose, but rather on the capacity of the regulatory subsystems within these cells to sense the energetic state of the intracellular metabolism.

As discussed in the previous Section, the regulatory subsystem includes two fundamental functional parts: the second-order constraints and the sensitive constraints (Figure 2B). From the organizational perspective, the elaboration of the model should therefore identify the parts that perform these functions. What are the sensitive constraints in this case? When glucose circulating in the blood is transported into beta-cells, it is metabolized and transformed into ATP. The increase in the ratio between ATP and ADP triggers a sequence of changes in some of the constraints in the cells (ion channels and membrane polarity), starting from the closing of ATP-sensitive K^+^-channels and the consequent depolarization of the membrane. The depolarization of the membrane is followed by the opening of voltage-dependent calcium ions channels. The channels opening activates the proteins of the SNARE^17^ complex of the membrane, which in turn controls the fusion of the membrane and the vesicles containing insulin, with the consequent release of this hormone in the bloodstream (see for example Röder et al., 2016). We submit that the channels are the best candidate to be understood as the sensitive constraints of the glycemia regulatory subsystem: they convert a quantitative change in the cellular metabolism (increased production of ATP) – induced by an increase of glycemia – into a qualitative change in the regulatory subsystem (the closing of the K + -channels and the depolarization of the membrane)^18^. From this point on, the activation state of the regulatory subsystem results into the release of the second-order constraint (insulin) through a series of intermediate steps involving changes in potential. Glucagon secretion by pancreatic alpha-cells is activated in a similar way through the calcium-mediated fusion of glucagon-containing vesicles with the cell membrane, controlled by the SNARE complex. Yet, activation in alpha- and beta-cells is triggered by opposite glucose concentrations because of the specificity of their sensitive constraints (the ion channels). In alpha-cells, activation is triggered by cAMP^19^ when glucose concentration, and consequently that of ATP, is low (Gaisano et al., 2012).

With regard to the second-order action of the regulatory constraints, insulin plays three functional roles: (1) it facilitates glucose uptake into cells of muscle and adipose tissues, (2) it stimulates glycogenesis and (3) it inhibits the release of glucagon by pancreatic alpha-cells and, consequently, glycogenolysis. The first function is exerted by facilitating the migration of the glucose transporter GLUT4 to the plasma membrane by stimulating the glucose transport/hexokinase (GT/HK) first-order constraints. The second function is exerted by directly controlling those first-order constraints responsible for the process of production of glycogen from glucose through the phosphorylation of the enzyme glycogen synthase (GSase). The third function of insulin is to inhibit the production of glucose from glycogen (glycogenolysis) by liver cells, by activating phosphoprotein phosphatases 1 (PP1), which dephosphorylate glycogen phosphorylase, thus inhibiting its activity (Hye-Sook et al., 2016), and by inhibiting the release of glucagon by alpha-cells. The later effect on alpha-cells occurs by modifying one of the first-order constraints involved, the membrane potential of alpha-cells (see for example Quesada et al., 2008).

The secretion by alpha-cells of glucagon, the other second-order regulatory constraint, is markedly increased when glycemia is low. In this circumstance insulin release is strongly decreased, and the intracellular ATP concentration in alpha-cells is low, while cAMP concentration is increased. Glucagon triggers a cascade of phosphorylation in liver cells, activating the enzymes (first-order constraints) responsible for glycogenolysis, which is the transformation of glycogen back to glucose. In addition to the increase of glucagon secretion triggered directly by hypoglycemia, there is a counterintuitive increase of glucagon triggered by hyperglycemia. This effect manifests spontaneously when there is a severe loss of beta-cells in diabetes type-I, because insulin secreted in paracrine fashion normally inhibits, as mentioned, the release of glucagon by the alpha-cells adjacent to beta-cells. However, it has been recently postulated that prior to the destruction of beta-cells in diabetes type I an increased glucagon concentration is observed and is attributed to hyperglycemia. Such an effect of hyperglycemia on glucagon release could be obtained experimentally in normal animals infused with glucose to obtain a chronic hyperglycemia (Jamison et al., 2011). Hence, the paradoxical induction of glucagon secretion is due to direct effects of hyperglycemia in the alpha-cell (Knudsen et al., 2019) and it is exacerbated indirectly by loss of beta-cells, when the inhibitory paracrine effect of insulin is lost.

A simple feedback model cannot adequately capture the two effects of hyperglycemia underlying the paradoxical increase of glucagon secretion (Wang et al., 2015), because it is centered on negative control loops between glycemia and two pancreatic hormones, insulin and glucagon. As a consequence, the feedback model makes organismal hierarchies collapse. This concerns all constraints from the second-order (as insulin and glucagon), to the higher-order constraints– such as the intra-islet interactions that regulate glucagon secretion, including beta-cells constraining glucagon secretion by the alpha-cells. The more orders of constraints are identified, the more the model *flattens* the functional organization of the organism (see for instance Röder et al., 2016, p. 5) the more the model loses explanatory adequacy. In fact, the feedback loop is useful to depict the situation in diabetes as long as the disease is seen as a problem due to lack of insulin (type 1 diabetes, T1D) or to insulin resistance (type 2 diabetes, T2D). However, since the discovery of the role of beta-cells on suppressing glucagon secretion by alpha-cells, the centrality of insulin deficiency on the genesis and maintenance of diabetes was re-evaluated, if not contested. As a result, the role of excess glucagon is given equal importance if not supremacy over the classical insulin centered view (bi-hormonal hypothesis). Intra-islet paracrine regulation of glucagon by the beta-cells, as well as by paracrine secretion of additional pancreatic hormones and by the nervous system are higher-order order constraints that are not represented *as such* in the feedback model^20^.

As discussed in the previous Section, regulation consists of the capacity to selectively modulate the first-order self-maintaining regime in response to specific variations of the internal and external environment, due to the action of a dynamically decoupled dedicated control subsystem that is sensitive to these variations. Regulation allows the new first-order regime to cope with the changed environmental conditions and internal requirements. Constraints exerted by alpha- and beta-cells from the pancreas comply with this characterization of regulation, by constituting a subsystem that is sensitive to specific conditions and exerts second-order control upon first-order constraints in a way that is dynamically decoupled from them. More specifically, we have identified seven sets of processes, seven sets of associated first-order constraints, and a regulatory subsystem endowed with two sets of second-order constraints. Depending on the capacity of some of the constraints of the regulatory subsystem to sense variations in the metabolic state of pancreatic cells, which in turn is affected by the supply of glucose, the system can selectively modulate the regime of first-order constraints: when perturbed by food intake, it does so through insulin, resulting in an increased production of glycogen from glucose; in the case of high energy demands, through glucagon, resulting in an increased production of glucose from glycogen. The overall result is the self-maintenance of the organism in varying conditions, achieved in particular through the homeostasis of glucose concentration in the plasma. While models relying on feedback loops only describe the relations between the variables involved in homeostasis, models relying on organizational closure can also *derive* these relations from the underlying functional organization of the organism. It is for this reason that, we hold, the organizational framework has a potentially higher explanatory power.

The above schema, however, focuses exclusively on metabolism and, accordingly, is still the first step in a more elaborate description. Many other, non-metabolic factors, including the nervous system, the intestine, and adipose and muscle tissues also participate in the modulation of insulin and glucagon secretion in relation to other environmental or internal conditions (Röder et al., 2016)^21^. This means that second-order regulatory constraints acting on the first-order regime are not at the top of the hierarchy but can themselves be modulated by higher-order constraints belonging to regulatory subsystems sensitive to different variables (Figure 2C). Unlike the feedback model, the organizational framework can naturally handle these additional regulatory subsystems by including in the closed graph the pertinent processes and constraints, and by making explicit the different orders involved. The procedure applied so far to identify and integrate first- and second-order functional constraints can be further iterated to obtain a richer and more adequate representation of the biological organization involved in the phenomenon under scrutiny.

## Discussion and Conclusion

Let us compare the two different descriptions of glucose regulation as represented in Figures 1, 2: feedback loops vs. organizational closure.

The representation in terms of feedback loops – and more generally in terms of single level networks – explains glucose homeostasis by focusing on the values of three variables, i.e., the concentrations of glucose (glycemia), insulin and glucagon. The relationships between these variables are determined by a set of functional components, which are included in the system as *independent* objects, i.e., as objects whose conditions of existence do not depend on the dynamics they control. As a result, feedback loops only include those relations among components – usually described as signals – that are relevant to explain the values of the target variables.

The main motivation behind the adoption of the organizational framework is radically different from that underlying feedback loops. Rather than focusing only on a specific relationship between variables, it also aims at understanding glucose homeostasis as a manifestation, as a consequence of the distinctive biological capacity of self-maintenance and, in particular, of organisms’ capacity to manage energetic resources. Accordingly, functional components contribute to the maintenance of a biological organization that, in turn, contributes to maintain their own conditions of existence. In the organizational terminology, functional components are subject to closure. Understanding glucose homeostasis in light of biological self-maintenance leads to a significant enrichment of the quantity and kinds of functional objects and processes involved. In particular, we have emphasized the crucial distinction between different *orders* of functionality, each of them implying different kinds of constraints and processes. The regulation of glucose concentrations and metabolism is achieved through the coordinated activity of a *hierarchy* of functional regimes, which seem to be overlooked by descriptions appealing to feedback loops.

While feedback loops rely on the machine analogy, organizational closure emphasizes the *dis*analogy. Most of the differences between the two frameworks, we submit, derive from this central epistemological divergence. But what is at stake is not just a matter of difference. It is our contention that the organizational framework possesses a higher explanatory power than descriptions in terms of feedback loops, insofar as the former can replace the latter, *but not vice-versa*. As we suggested with the example of glucose regulation, the organizational framework can explain the homeostasis of a variable as the result of the functional and hierarchical complexity of an organism. In contrast, a description in terms of feedback loops cannot capture the functional complexity of the organization underlying biological homeostasis. There are many ways to realize the same feedback loop, and the organizational framework aims at understanding how biological organisms achieve that goal in each specific circumstance (e.g., through coupled processes, loops involving one order of constraints, regulatory loops involving second-order constraints, higher level regulatory loops including third order constraints and so on and so forth) and explaining its functional significance. In a word, we argue that organizational models can explain all what feedback models explain, and *more*.

The higher explanatory power of the organizational perspective has further implications, that allow going beyond the weaknesses of feedback loops mentioned at the end of Section “The Standard Representation of Glycemia Regulation: The Feedback Loop.” A description of regulation in terms of organizational closure, by placing it in the context of the organism, enables the ascription of several functions to the same component, or the ascription of a given function to a set of distributed structures or even – to refer again to the “device” that sets the normal range of a target variable – to the organism as a whole. The different posture with respect to an engineering conception that describes a phenomenon by appealing to a system of fixed components and localized and univocal functional roles appears clearly here. Also, unlike the feedback description, the organizational perspective fosters the progressive integration of additional processes and functional constraints into the description, insofar as its main explanandum is not the homeostasis of a variable *per se*, but the capacity of self-maintenance of the organism as a whole. In particular, it encourages improving the description of a system by specifying several hierarchical orders of regulatory constraints.

Another important implication, already evoked at the end of Section “The Standard Representation of Glycemia Regulation: The Feedback Loop,” is that the organizational framework leaves room for variations in the regulation of glucose (as well as other variables) and departures from a given range of homeostatic values. The reason, again, is that glucose homeostasis is not a goal in itself, but a means to achieve self-maintenance; accordingly, an organism can vary its behavior as soon as the specific internal and external conditions require an adaptive response, be it a temporary or irreversible shift. The difference with regulation in machines, which are designed *for* maintaining a variable within a given range (deviations from the latter being therefore conceived as an error), is blatant here. Lastly, and symmetrically with respect to the previous point, the understanding of homeostasis from an organicist perspective opens the possibility of connecting homeostasis with biological norms, and thus with judgments about health and disease, while feedback loops cannot. Just as deviations from the normal range are not necessarily bad, the maintenance of homeostasis is not necessarily good: their biological significance depends on the general state of the organisms and its adaptive needs. Fever in response to infection is one of the best-known adaptive responses, as is the stress response. The recent availability of instrumentation to perform continuous glucose monitoring also revealed that normoglycemic individuals (according to standard clinical measurements) exhibit high interstitial glucose concentration variability; their glucose concentrations may reach prediabetic and diabetic ranges during a significant portion of the monitored time. This interindividual variability also appears as a response to standardized meals, thus suggesting that glucose homeostasis within a specific range is not as “normal” as previously thought (Hall et al., 2018). In this regard, it is useful to think on the adaptive potential of interindividual variability. For example, insulin resistance is considered by some as an adaptive response that protects the cardiovascular tissues from nutrient-induced injury, rather than the main culprit of the T2D syndrome (Nolan et al., 2015).

Because of these implications, we argue that the organizational framework is worth exploring. The representation of glycemia regulation offered here constitutes what is sometimes called a “proof of concept,” i.e., an illustration of how the framework could apply to a specific biological phenomenon and how it can be represented through diagrams^22^. Its epistemological role consists in fostering the elaboration of more precise organizational *models*, which would make explicit many other aspects, starting with the times scales and the topology of the constraints involved in the control and regulation of the relevant processes. One recent example of a model relying on organizational principles is provided by the work of Montévil and coworkers on mammary organogenesis (Montévil et al., 2016).

To be sure, the scientific fruitfulness of the organizational framework can be adequately assessed (as is the case for any theoretical proposal) only by looking at the quantity and quality of the models that it could generate. Yet, the illustration provided here has the merit of making explicit some guidelines for elaborating models, which should focus – as discussed in Section “Reinterpreting Glucose Regulation from Organizational Principles” – on those objects that play a central role in the organizational framework: processes, time scales, constraints, constraint dependencies, closure and the hierarchy of functional orders. It also shows that the organizational framework tends to promote the integration into a single model of experimental data that are usually obtained and exploited separately by different experimental groups and scientific communities (see also Veloso, 2017). Accordingly, the framework can make an important contribution in overcoming the compartmentalization that sometimes characterizes experimental research. Conversely, the inherent tendency to integrate data into organism-centered models may induce the search of new experimental data required to “bridge the gap” between aspects and phenomena that have never been treated and interpreted jointly.

The proof of concept provided in this study also fulfills another epistemological function, which consists of putting some of the challenges that an organization-centered modeling strategy has to face into the foreground.

One challenge consists of integrating a variety of processes, interactions and associated functional constraints realizing organizational closure, by making their topological and quantitative relations explicit. Insofar as they would explore new ways of looking at biological organisms, organizational models may require developing original formal and mathematical tools. Moreover, the various levels and orders that constitute the hierarchical organization of the organism should be discriminated and integrated. However, the task of drawing the functional boundaries of a given regulatory subsystem, by identifying both its sensitive and higher-order constraints, may prove to be difficult to achieve in some cases. What is at stake here is the specific nature of biological organisms, whose functional organization is the result of an ontogenetic process (instead of an assemblage of pre-existing parts, as in machines) and an evolutionary history.

The organizational framework also faces the reciprocal challenge vis-à-vis complexity. A satisfactory model does not need to include all the details about the processes and constraints at play in the organism: a trade-off between precision and comprehensiveness must be found. The proof of concept presented here shows that an organizational framework can provide theoretical guidelines for locating a target phenomenon in the overall organization, and for removing dispensable aspects. The organizational diagram given above, for instance, focuses on various constraints that play a direct role in the regulation of glycemia and, in turn, neglect other constraints involved in the metabolism. The possibility of simultaneously detailing some functional aspects while bracketing others allows the framework to make the model more relevant with regards to the specific phenomenon under scrutiny, while maintaining the general characterization of closure. More precisely, the fact of detailing or neglecting specific aspects is achieved by “zooming” in or out when describing the constraints and the processes on which they act. In the above diagram, for instance, constraints such as insulin, glucagon, alpha- and beta-cells and their target processes are described in a more detailed way when compared to the digestive system or the vascular system. Also, the framework allows neglecting details at some level of description, when it focuses on processes and functions located at other levels. For example, when considering a tissue as a constraint performing a function (and not as a collection of cells), one may concentrate on collective aspects like cell junctions, cell adhesion to the substrate and cell–matrix interactions, and ignore – at least to some extent –intracellular aspects and components.

As a result, even though the organizational diagram includes more objects than the feedback loop, it succeeds in providing a tractable and useful description of biological complexity. Most of the time, the decision about which functional aspects should be detailed and which one should be bracketed depends on the phenomenon being considered and the explanatory aim of the model. Yet, the organizational framework might also be able to elaborate some general guidelines in this respect: exploring this question could elicit a fundamental epistemological discussion on modeling practices from an organicist perspective.

## Author Contributions

All authors listed have made an equal contribution to the work, and approved it for publication.

## Conflict of Interest

The authors declare that the research was conducted in the absence of any commercial or financial relationships that could be construed as a potential conflict of interest.

## Footnotes

1 By ‘distinctive complexity’ we mean those aspects that are *specific* to living systems, notably linked to their functionality (Longo et al., 2015). It refers to their proper “way of being.” Understanding biological complexity is therefore a central task of biological research. Complexity should not be confused with notions as ‘complication,’ which has a rather negative connotation and refers to aspects that might be difficult to access theoretically or experimentally, or both.

2 By ‘asymmetry’ we mean here that control is not a mutual interaction between entities, but a relation in which it is possible to distinguish between what acts and what is acted upon.

3 By ‘degree of freedom’ we mean the number of variables of a process that are free to vary independently.

4 The organizational framework considers that the hierarchies of *levels* and *orders* are distinct. A ‘level’ designates a closed regime of constraints that is either made of constituents, or included in an encompassing system, realizing themselves a distinct closure. An ‘order,’ in turn, designates a kind of functional object that operates over (modulates) other functional objects, within the same regime of closure. See Moreno and Mossio (2015, p. 143) for details.

5 That is, in terms of quantitative relationships between different compounds involved in chemical reactions.

6 For example, diauxic shifts in the metabolism of bacteria between different carbon sources, regulated by switching the genes involved in the synthesis of the relative enzymes on and off.

7 Autonomous agency can be defined as the set of activities that modify the environment of the system that generates them and are “performed according to a certain goal or norm” (Moreno, 2018, p. 290), which is related to the maintenance of the system itself.

8 See Bich and Moreno (2016) for an analysis of bacterial chemotaxis in terms of regulatory constraints.

9 For a discussion of the importance of regulation to understand life and its origins, see also Cornish-Bowden and Cárdenas (2019).

10 An example of a second-order constraint is a kinase enzyme catalyzing the phosphorylation of another enzyme in the system, leading to the activation or inhibition of the latter, while being locally unaffected by such interaction (for example, the kinase is not consumed in the reaction).

11 In this framework, biologically relevant entities can have a twofold role. A molecule, for instance, can be characterized as a *stimulus* when, by interacting with a sensitive constraint, induces the activation of the latter. The same molecule, instead, can play the role of a *metabolite* when consumed as a substrate in a metabolic process.

12 This is exemplified by the kinases. Their activation is triggered by an interaction on a different site than the active one that catalyzes the phosphorylation of other enzymes. In this case, the regulatory subsystem is realized by a single enzyme. In other cases, as we will discuss, the subsystem is constituted by a set of different constraints playing different roles, and collectively realizing the regulation.

13 On the biological importance of control constraints that exhibit context-sensitive activity, see also Pattee (1972); Abel (2010), and Winning and Bechtel (2018).

14 Veloso (2017) provides a mathematical approach to detect regulatory decoupling in the case of epigenetic control during the development of multicellular systems.

15 Gluconeogenesis (process number 6) contributes to this balance during fasting, exercise and when other sources are mostly depleted.

16 Variations in glucose concentrations can only change glycogen production linearly in the absence of a significant release of insulin (Schafer et al., 2005).

17 SNARE (SNAP receptor) is an acronym designating a group of proteins that mediate membrane fusion and exocytosis. SNAP is an acronym for Soluble NSF (*N*-ethylmaleimide-sensitive factor) attachment proteins.

18 It is important to point out that glucose is part of the metabolic process constrained by first-order constraints, in this specific case the enzyme glucokinase, which catalyzes the same process in a multitude of different cell types. The sensitive constraint belonging to the regulatory subsystem should not be part of the process or the first-order constraint, but should bring forth a qualitative change. In our proposal this corresponds to the ion channel. Regulation is activated in beta-cells with the polarization of the membrane, a qualitative change that is not metabolic anymore and which for example does not take place in other types of cells.

19 Cyclic adenosine monophosphate (cAMP), obtained from ATP, is a second messenger involved in various physiological processes. The activation of glucagon secretion in alpha-cells is triggered by cAMP when ATP concentration is low.

20 Another interesting example of therapeutic effects that are not easily explained by the feedback loop is the remission of T2D by bariatric surgery. Although this procedure was originally used to reduce weight in obese patients, it was found that it also induced remission of T2D. In particular, this procedure revealed the importance of the small intestine in glucose homeostasis. Several hypotheses were advanced on the importance of each of the components underlying remission, from glucose and nutrient sensing (sensitive constraint) to incretin secretion (third order constraint) (Laferrère, 2016). While these functions could be integrated into a hierarchical organizational description, they would suffer from the same problem of flattening in a feedback loop.

21 The fact that alpha- and beta-cells are sensitive to their internal state rather than directly to glucose, makes it possible for distinct processes (not directly dependent on glucose) to coordinate different steps of the activation process of insulin and glucagon secretion. For example “insulin release is stimulated by the so-called cephalic phase, which represents the conditioned reflex of increased hormone secretion, referred to as cephalic phase insulin response, even in the absence of nutrients/glucose as a trigger, such as when anticipating a meal, to prepare the organism to adequately respond to incoming nutrients”(Röder et al., 2016, p. 5).

22 As argued by Bechtel (2008), diagrams are an important component of scientific reasoning, useful in order to represent how the phenomenon under study is generated through the ‘well-orchestrated’ organization of the components that participate in it. Diagrams are needed precisely to develop a reasoning that captures both the operations performed by the parts and the relationship between these operations.
